# Chenodeoxycholic Acid Enhances the Effect of Sorafenib in Inhibiting HepG2 Cell Growth Through EGFR/Stat3 Pathway

**DOI:** 10.3389/fonc.2022.836333

**Published:** 2022-02-17

**Authors:** Yang Zhang, Yan Zhang, Xiao-Jun Shi, Jun-Xiang Li, Lin-Heng Wang, Chun-E Xie, Yun-Liang Wang

**Affiliations:** ^1^ Department of Gastroenterology, Dong Fang Hospital, Beijing University of Chinese Medicine, Beijing, China; ^2^ Graduate School, Beijing University of Chinese Medicine, Beijing, China

**Keywords:** chenodeoxycholic acid, sorafenib, liver cancer, combination therapy, epidermal growth factor receptor

## Abstract

**Background:**

Hepatocellular carcinoma (HCC) is a highly invasive disease with a high mortality rate. Our previous study found that Chenodeoxycholic acid **(**CDCA) as an endogenous metabolite can enhance the anti-tumor effect. Sorafenib has limited overall efficacy as a first-line agent in HCC, and combined with CDCA may improve its efficacy.

**Methods:**

HepG2 cells and Balb/c nude mice were used respectively for *in vitro* and *in vivo* experiments. Flow cytometry, Western blotting, HE and immunohistochemical staining and immunofluorescence were used to study the effects of CDCA combined with sorafenib on HepG2 cell growth and apoptosis-related proteins. Magnetic bead coupling, protein profiling and magnetic bead immunoprecipitation were used to find the targets of CDCA action. The effect of CDCA on EGFR/Stat3 signaling pathway was further verified by knocking down Stat3 and EGFR. Finally, fluorescence confocal, and molecular docking were used to study the binding site of CDCA to EGFR.

**Results:**

In this study, we found that CDCA enhanced the effect of sorafenib in inhibiting the proliferation, migration and invasion of HepG2 cells. Magnetic bead immunoprecipitation and protein profiling revealed that CDCA may enhance the effect of sorafenib by affecting the EGFR/Stat3 signaling pathway. Further results from *in vitro* and *in vivo* gene knockdown experiments, confocal experiments and molecular docking showed that CDCA enhances the efficacy of sorafenib by binding to the extracellular structural domain of EGFR.

**Conclusion:**

This study reveals the mechanism that CDCA enhances the inhibitory effect of sorafenib on HepG2 cell growth *in vitro* and *in vivo*, providing a potential new combination strategy for the treatment of HCC.

## Introduction

Hepatocellular carcinoma (HCC) is a highly invasive disease with high mortality and the fourth leading cause of cancer-related deaths worldwide. Although the understanding and treatment of HCC have greatly improved in the past decades, HCC remains one of the malignant tumors with the worst prognosis (1). Surgical resection, transplantation, ablation, transarterial chemoembolization, and targeted therapy have been proven to be beneficial to survival; however, to date, these therapies are unable to effectively reduce morbidity and mortality, and the five-year survival rate of patients with HCC remains low (The average five-year survival rate of HCC patients in the US is 19.6%) (2).

Sorafenib is the first-line US Food and Drug Administration (FDA)-approved drug for liver cancer; it can extend the median survival of patients from 7.9 months to 10.7 months (3, 4). Unfortunately, only a small number of patients respond to sorafenib, and its serious side effects often lead to dose reduction or treatment discontinuation (5). Thus, there is an urgent need for more effective treatment strategies for liver cancer. Studies exploring sorafenib combination strategies have revealed several effective synergistic drugs. Capsaicin, Silibinin, and panobinostat were able to improve the efficacy of sorafenib to varying degrees, but were still unsatisfactory. Therefore, there is a need to find more effective combination drugs (6–8).

Bile acids metabolism plays an important role in health, and some studies have found that bile acids are associated with HCC progression (9). Chenodeoxycholic acid (CDCA) is one of the major primary bile acids in human and animal bile (10), which had been applied in the clinical treatment of cholesterol gallstones for a long history (11). Due to the side effects of diarrhea, CDCA has been gradually replaced by ursodeoxycholic acid (UDCA) in cholesterol gallstones treatment (11). However, a previous study found significantly lower levels of CDCA in serum and tissue samples from patients with hepatocellular carcinoma, suggesting that CDCA may be a hepatocyte protective agent (12). Although our colleagues have reported that CDCA may be a carrier of liver tumor-targeting drugs, this finding has not been taken seriously (13). In our previous study (unpublished data), we found that CDCA can be effectively implemented in anti-tumor therapy (patent No. ZL 2017 1 0225207.2; patent application No. 202110185336.X). It is necessary to elucidate the target of CDCA as a target drug carrier for hepatocellular carcinoma, and this paper verified that CDCA is enhancing the therapeutic effect of sorafenib on hepatocellular carcinoma through EGFR/Stat3 pathway.

## Materials and Methods

### Cell Culture

HepG2 cells were purchased from Guangzhou Cellcook Biotech Co.,Ltd (Guangzhou, China) and the cell line was authenticated by operator Xiaohua Mo from Guangzhou Cellcook Biotech Co.,Ltd. Frozen cells were recovered in a water bath at 42°C within 1 min and transferred into T-25 culture flasks for culture. The cells were maintained in RPMI 1640 medium (GIBCO, Cat. No.11875093, Thermofisher scientific, China) containing 10% fetal bovine serum (GIBCO, Cat. No.12664025, Thermofisher scientific, China) in a humidified environment at 37°C.

### Cell Proliferation Assay

After the drug interventions, the culture medium was replaced with 100 μL of serum-free medium and 10 μL of MTT solution (Cat. No.V13154, Thermofisher scientific, China) and the cells were further incubated at 37°C for 4 h. The reaction solution was discarded and the cells were resuspended with the cell solution containing 10% sodium dodecyl sulfate (SDS). To measure cell proliferation, the absorbance 490 nm was read using a spectrophotometer (Perlong, DNM-9602, China).

### Cell Scratch Assay

After 24 h of cell culture, a single-cell suspension was prepared with Dulbecco’s modified Eagle’s medium (DMEM, GIBCO, Cat. No. 11965092, Thermofisher scientific, China) with 10% FBS at 1 × 10 ^5^ cells/mL. The cells were seeded in 6-well plates and cultured until confluence. Then, a scratch was created with a sterile 200-μL pipette tip. Cell migration was monitored at 0 h, 24 h, and 48 h using a digital camera system. The relative wound width was calculated as (final diameter width)/(original diameter width). The experiment was repeated three times.

### Transwell Chemotaxis Assay

A serum-free cell suspension (100 µL) was added to the upper Transwell chamber. Five hundred microliters of DMEM containing 10% FBS was added to the lower chamber. The Transwell chambers were incubated at 37°C in the presence of 5% CO_2_ for 24 h. Migrating cells on the lower side of the membrane were fixed with 4% paraformaldehyde and stained with 0.1% crystal violet solution for 15 min. The number of invading cells was counted under a light microscope (Chongqing Photoelectricity, XDS-2B, China).

### Flow Cytometry

After the drug interventions, cells were washed with PBS (Cat. No. 20012050, Thermofisher scientific, China) three times for 5 min each time and resuspended in 100 μL of 1× binding buffer. Then, 10 μL of Annexin V-FITC (BD, Cat. No. 556547) and 5 μL of PI (Roche, Cat. No.11697498001) were added and the cells were incubated at room temperature in the dark for 30 min. The cells were centrifuged at 1,000 rpm for 5 min and the precipitates were resuspended in 500 μL of 1× binding buffer and detected on a flow cytometer (BD, Callibure) (FL1-H for Annexin V-FITC fluorescence channel and FL2-H for PI fluorescence channel). The data were analyzed using CellQuest software (BD CellQuest Pro, RRID : SCR_014489).

### *In Vivo* Study

Four-week-old female Balb/c nude mice (specific pathogen-free grade) were provided by Charles River (Beijing, China). All mice were housed in a pathogen-free, temperature- and humidity-controlled environment with laminar air flow under a 12-h day/night cycle. The mice had free access to food and water. Prior to the start of the study, the mice were acclimatized for one week. Then, the mice were injected subcutaneously in the right abdominal wall with 1 × 10^7^ HepG2 cells in 200 μL of sterile phosphate buffer. Tumor growth was observed daily and at a tumor volume of approximately 80 mm^3^ the mice were divided into four groups (n = 5 per group) randomizely and treated by daily gavage. The control group was given 200 μL of saline, the CDCA group was given 30 μg CDCA/200 μL (Qingdao Jieshikang, Cas: 474-25-9, China), the sorafenib group was given 40 μg sorafenib/200 μL, and the combination group was given CDCA 30 μg + sorafenib 40 μg/200 μL for 14 consecutive days. Tumor volume and body weight were measured every other day. At the end of the study period, all mice were executed by cervical dislocation under pentobarbital sodium (50 mg/kg) intraperitoneal anesthesia, and tumor specimens were collected. All animal experimental procedures were performed in accordance with the guidelines of the Good Laboratory Practice (No. CNAS GLP0023) and complied with the relevant requirements of the Experimental Animal Ethics Committee (Approval No. 2020041501) of CAIQ Health (TianJin) Inspection and Testing Co., LTD.

### Preparation of the Magnetic Beads-CDCA Complex

NHS-magnetic beads (Cat. No. 88826, Thermofisher scientific, China) were selected based on the active group in the chemical structure of CDCA. After washing, an equal volume of CDCA was added to the beads and the mixture was left to stand at room temperature for 1 h. After incubation at 4°C for 1 h, the supernatant was collected. Two volumes of blocking buffer were added and the beads were left to stand at room temperature for 2 h. Finally, an equal volume of 1× PBS was added and mixed thoroughly, and the beads were stored at 4°C.

### Western Blotting

Western blotting analysis was performed as previously described (14). Proteins were isolated using the RIPA method. After adjusting the protein concentration, the proteins were separated by SDS-polyacrylamide gel electrophoresis (PAGE) and transferred to polyvinylidene difluoride membranes using an eBLOT system. The membranes were incubated with primary antibodies diluted in TBS with 5% skimmed milk powder. The primary antibodiecs p53 (2524, Cell Signaling Technology, Danvers, MA, USA), caspase 3 (ab2171, Abcam, Cambridge, UK), Stat3 (12640, Cell Signaling Technology), p-Stat3 (9145, Cell Signaling Technology), EGFR (2232, Cell Signaling Technology), p-EGFR (3777, Cell Signaling Technology), and GP130 (ab226346, Abcam) and second antibodies Goat anti-rabbit IgG(H+L), HRP (Jackson ImmunoResearch, Cat. No.111035003), Goat anti-mouse IgG(H+L), HRP (Jackson ImmunoResearch, Cat. No.115035003) were used. The membranes were developed using ECL for 3–5 min.

### Kaomas Brilliant Blue Staining

Total protein extract (100 μL) was mixed thoroughly with an equal volume of magnetic beads and incubated at 4°C for 30 min. The beads were separated by magnetic racking for 1 min and the supernatant was collected. Protein samples after magnetic bead sorting, supernatant samples after sorting, and total protein were subjected to SDS-PAGE electrophoresis. Bound protein was confirmed by Kaomas brilliant blue staining.

### Mass Spectrometry

After separation by SDS-PAGE, proteins were excised from the Kaomas brilliant blue-stained gel, decolorized, and lyophilized. Then, 40 μL of trypsin buffer (Cat. No. 25200072, Thermofisher scientific, China) was added and the mixture was incubated at 37°C for 16–18 h. The proteins were separated by capillary high-performance liquid chromatography and analyzed by mass spectrometry using a Q Exactive mass spectrometer (Thermo Scientific). Proteins were identified by comparing the determined molecular weights with the theoretical peptide masses of proteins registered in the UniProt/NCBI database.

### Magnetic Bead-Immunoprecipitation

The homogenized specimen was added with PBS at a ratio of 1 ml PBS/10^7^ cells, along with protease inhibitor, resuspended, and the supernatant was collected by incubation on ice for 30 min and then centrifuged at 2000 rpm for 20 min. Protein quantification was performed by BCA method. 200ug of total protein extract was mixed thoroughly with an equal volume of drug-magnetic beads, incubated at 4°C for 60 min, and the beads were separated by magnetic rack for 1 min and the supernatant was collected as IP supernatant. The protein-CDCA-magnetic beads were separated by magnetic rack, and the protein-CDCA-magnetic bead precipitate was re-solubilized with SDS electrophoresis loading buffer.

### Gene Knockdown

Cells were digested using a conventional method and the cell density was adjusted to 1 × 10^6^/mL. The cells were seeded in 6-well cell culture plates (1.5 mL/well) and cultured for 24 h. Two micrograms of plasmid was added to and mixed with 100 μL of serum-free medium to obtain solution a; 4 μL of Lipofectamine 2000 was added to and mixed with 100 μL of serum-free medium to obtain solution b; solutions a and b were mixed and left at room temperature for 15 min. Then, the medium was replaced with 800 μL of serum-free medium and solutions a and b were added slowly to the cells. After 4–8 h, the medium was exchanged, and the cells were collected after 48 h and used for western blotting as described above to detect the expression of EGFR and Stat3 to confirm effective knockdown and obtain stable cell lines. Cells were spread in 96-well cell culture plates 24h after transfection, and growth medium containing 2μg/ml Puromycin was added 48h after transfection and cultured continuously for more than 8 weeks. The cells were collected and the expression of EGFR and stat3 was detected by WB method (same method as before) to confirm the stable cell lines.

### Confocal Microscopy

Cells were incubated with magnetic beads-CDCA for 15 min, washed, and incubated with 150 μL of medium containing EGFR antibody at 37°C for 1 h. The cells were incubated with goat antibody rabbit IgG-AF488 antibody (SouthernBiotech Cat. No. 0121-30,RRID : AB_2794062) in the dark at room temperature for 30 min. DAPI working solution was added and the cells were further incubated in the dark at room temperature for 15 min. EGFR expression and magnetic bead binding were observed by confocal microscopy (Nikon, C2+).

### Histological and Immunohistochemical Analyses

Freshly collected tumor tissues were fixed in 4% paraformaldehyde, paraffin-embedded, and sectioned. The sections were stained with HE. For immunohistochemistry, the section were deparaffinized and subjected to heat-induced antigen unmasking. EGFR and STAT3 antigens were used. Images were acquired with an OLYMPUS microscope.

### Immunofluorescence Staining

Paraffin sections were dewaxed, rinsed with gradient alcohol, and placed in 0.1 mol/L citrate repair solution (pH 6.0) for antigen repair. After washing, the tissues were circled with an immunohistochemical pen and incubated with a drop of 5% goat serum at room temperature for 1 h. Two primary antibodies at appropriate concentrations were added dropwise, and a mixture of secondary antibodies [Coralite 594 goat anti-mouse IgG (H+L) (Proteintech, Cat. No. SA00013-3) and Alexa Fluor 488 goat anti-rabbit IgG (H+L)] (Jackson ImmunoResearch, Cat. No. 115-545-003) were added dropwise. The sections were incubated for 30 min at room temperature and washed. Nuclei were stained with DAPI (Erwan Pathology, Cat. No. ER201707132) that was added dropwise.

### Molecular Docking

EGFR protein 3D structures (ID:4UV7) were obtained from the PDB database (https://www.rcsb.org/), and CDCA molecular structures were downloaded from the pubchem database (https://pubchem.ncbi.nlm.nih.gov/). Water molecules, small molecule ligands and redundant chains were removed from the protein crystal structure using Pymol software and molecular docking was performed using the AutoDock Vina program in AMDOCK software. After the completion of docking, compounds and target proteins with the highest docking scores and stable conformations were selected for further visualization using Discoverstudio and Pymol software.

### Statistics

The statistics program ‘IBM SPSS Statistics 25’ is used for the statistical evaluation. Unless elsewhere stated, bars represent means ± SD. Statistical comparisons between two groups were conducted by the unpaired two-tailed t-tests. Statistical comparisons among multiple groups were conducted by one-way ANOVA tests and *post hoc* tests for the indicated comparisons. Statistical differences of *p* < 0.05 were considered significant.

## Results

### CDCA Enhances the Proliferation-Inhibitory Effect of Sorafenib on HepG2 Cells

**Figure 1:**
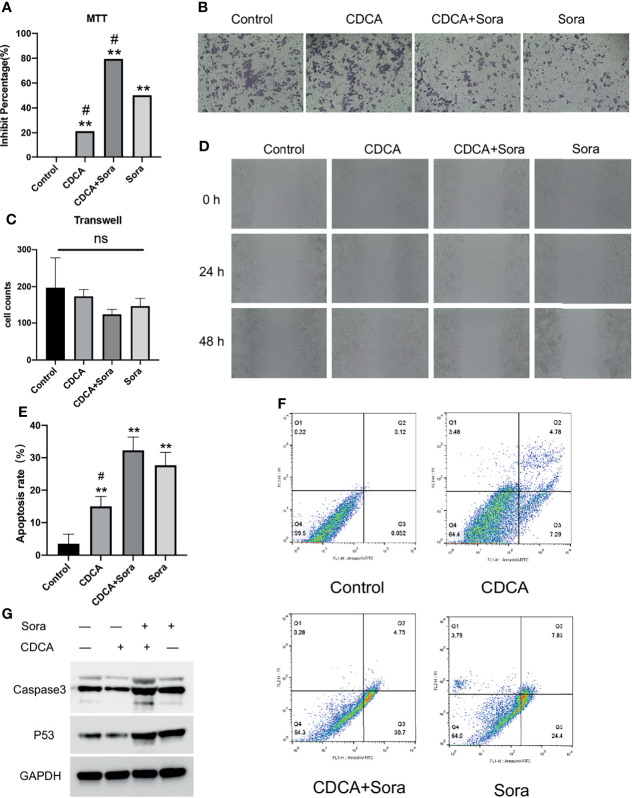
CDCA inhibits cells *in vitro* in synergy with sorafenib. HepG2 cells were treated with 10 μM sorafenib, 1 μg/mL CDCA, or 1 μg/mL CDCA plus 10 μM sorafenib for 24 hours. **(A)** MTT assay of cell proliferation in all treatment groups. **P < 0.01 vs. control group, ^#^ *P* < 0.05 vs. sorafenib group. **(B)** Crystalline violet staining to observe cell proliferation (20x). **(C)** Transwell assays revealed no significant difference between the groups. ns, no significance. **(D)** Representative images of scratch assay results at 0, 24, and 48 h (10x). **(E)** Apoptosis rates in all treatment groups. **P < 0.01 vs. control group, ^#^ *P* < 0.05 vs. sorafenib group. **(F)** Flow-cytometric detection of apoptosis in all treatment groups. **(G)** Western blot detection of cell supernatant caspase 3 and p53 expression levels.

To evaluate the potential effect of CDCA on the efficacy of sorafenib, we first treated HepG2 cells with 10 μM sorafenib (15), 1 μg/mL CDCA, or 1 μg/mL CDCA plus 10 μM sorafenib *in vitro* (Effective non-toxic concentration dose from normal cell screening, **Figure S1**) for 24 hours. Cell proliferation assay results indicated that CDCA combined with sorafenib had a stronger inhibitory effect on HepG2 cells than sorafenib alone (*p* < 0.05) (**Figure 1A**). The same results were shown in the SMMC 7721 cell line (**Figure S2**). Crystalline violet staining revealed a decrease in the proliferation of cells treated with the drug combination than other groups (**Figure 1B**).

### CDCA Plus Sorafenib Inhibits the Migration and Invasion of HepG2 Cells

The inhibitory effects of CDCA and sorafenib on HCC cell migration were investigated using cell scratch assays. As shown in **Figure 1D**, the migration of HepG2 cells was more strongly inhibited by the combination treatment than by any of the single-agent treatments. At 24 and 48 h, scratch wounds in both the sorafenib and combined treatment groups were wider than those in the control group. The results showed that CDCA and sorafenib had a synergistic inhibitory effect on the migration of HepG2 cells, whereas CDCA alone did not have a significant effect. Transwell assay results (**Figure 1C**) showed that CDCA plus sorafenib inhibited the invasion of HCC cells, although the differences with the other treatment groups were not statistically significant (*P* > 0.05).

### CDCA Enhances Sorafenib-Induced Apoptosis in HepG2 Cells

To evaluate whether CDCA enhances sorafenib-induced apoptosis in HCC cells, apoptosis after the treatments was examined by flow cytometry. The results showed that the combination treatment induced a higher level of apoptosis than any of the single-agent treatments (**Figure 1F**). After 24 h of treatment, the apoptosis rates were 3.53%, 15.017%, 27.68%, and 32.267% in the control, CDCA, sorafenib, and CDCA plus sorafenib group, respectively (**Figure 1E**). To elucidate the molecular mechanism of increased apoptosis in HepG2 cells, we used western blotting to analyze the expression of the apoptosis-related proteins caspase 3 and p53. CDCA plus sorafenib upregulated the expression of caspase-3 and p53 than any other treatment (**Figures 1G** and **S3**). These findings suggested that CDCA plus sorafenib regulates the activation of apoptosis signaling in HepG2 cells.

### CDCA Plus Sorafenib Inhibits the Growth of Transplanted HepG2 Cells *In Vivo*

**Figure 2:**
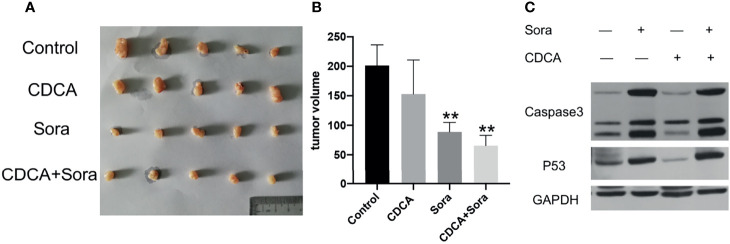
CDCA plus sorafenib inhibits tumor growth and promotes tumor cell apoptosis *in vivo*. Four-week-old female Balb/c nude mice were injected subcutaneously in the right abdominal wall with 1 × 10^7^ HepG2 cells in 200 μL sterile phosphate buffer. At a tumor volume of approximately 80 mm^3^ the mice were divided into four groups (n = 5 per group) and treated by daily gavage. The control group was given 200 μL of saline, the CDCA group was given 30 μg CDCA/200 μL (15mg/kg), the sorafenib group was given 40 μg sorafenib/200 μL (20mg/kg), and the combination group was given CDCA 30 μg + sorafenib 40 μg/200 μL for 14 consecutive days. **(A)** Photographs of tumors after transplantation of HepG2 cells into nude mice followed by drug gavage for 14 days. **(B)** Tumor size in all the treatment groups. **P < 0.01 vs. control group. **(C)** Western blot detection of apoptotic protein caspase 3 and p53 expression in tumor tissues.

To evaluate whether CDCA plus sorafenib can inhibit tumor growth *in vivo*, 1 × 10^7^/200 μL HepG2 cells were inoculated subcutaneously into the right abdominal wall of 4-week-old Balb/c nude mice. When the tumors reached a volume of approximately 80 mm^3^, the mice were divided into four treatment groups: CDCA 30 μg/200 μL (15mg/kg), sorafenib 40 μg/200 μL (20mg/kg) (16), CDCA 30 μg + sorafenib 40 μg/200 μL, and 200 μL of normal saline as a control. All treatments were administered for 14 days. CDCA plus sorafenib had a significantly stronger inhibitory effect on tumor growth than sorafenib alone, whereas CDCA had no significant effect (**Figures 2A, B**). The results of western blot analysis of apoptosis-related protein expression were consistent with those obtained *in vitro* (**Figures 2C** and **S4**). Together, the *in vitro* and *in vivo* results suggested that CDCA plus sorafenib effectively inhibits the growth, invasion, and metastasis of HCC cells, promotes tumor cell apoptosis, and shows better efficacy than sorafenib alone.

### CDCA Enhances the Effect of Sorafenib by Targeting HepG2 Cells

**Figure 3:**
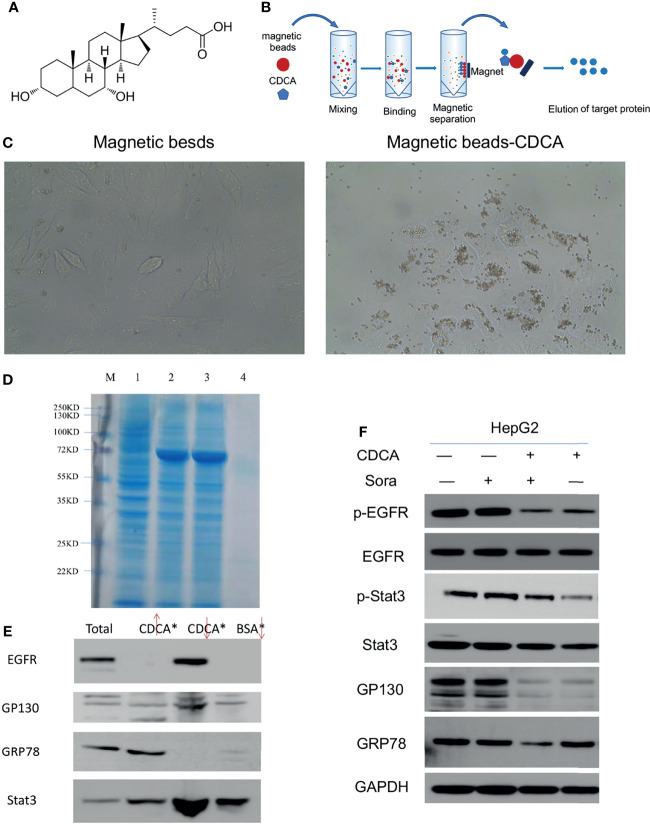
Targeting of CDCA to HepG2 cells. Using magnetic beads-CDCA coupling, total binding proteins were obtained by precipitation, and binding proteins were identified by mass spectrometry analysis. **(A)** Chemical structure of CDCA. **(B)** Schematic diagram of CDCA coupling to magnetic beads. **(C)** Light micrograph of CDCA magnetic beads coupled to HepG2 cells after co-culture (40x). **(D)** Kaomas brilliant blue staining of binding protein, M is Marker,1 is total protein,2 is CDCA magnetic bead isolate protein 1,3 is CDCA magnetic bead isolate protein 2,4 is BSA bead isolated protein. **(E)** Western blot detection of EGFR, GP130, GRP78 and Stat3 protein expression by magnetic bead-immunoprecipitation assays (From left to right, total protein, magnetic bead supernatant, magnetic bead precipitate, BSA control). **(F)** The cellular experiments in **Figure 1** were repeated to confirm the changes in protein levels after drug intervention.

The magnetic bead method is an effective technique for drug target research (17). To investigate the mechanism of the enhanced anti-tumor effects of CDCA and sorafenib further, we first carried out magnetic bead precipitation assays. Based on the chemical structure of CDCA (**Figure 3A**), suitable magnetic beads were selected to produce magnetic beads-CDCA complexes. HepG2 cells were cultured in the presence of magnetic beads alone or magnetic beads-CDCA (**Figure 3B**). The results revealed that magnetic beads-CDCA substantially aggregated around the HepG2 cells, whereas magnetic beads alone did not (**Figure 3C**). To verify the binding of magnetic beads-CDCA to HepG2 cells, we extracted the total protein and stained it with Kaomas brilliant blue. As shown in **Figure 3D**, we observed binding proteins, indicating that the aggregation of CDCA on HepG2 cells occurred through protein binding. This result suggested that CDCA sensitizes the cells to and potentiates the effect of sorafenib.

### EGFR/Stat3 Signaling Pathway May Be the Mechanism of CDCA Synergistic Sorafenib

Based on the binding protein size and mass spectrometry analysis of the isolated protein and database comparison, we found that CDCA may bind GRP78, Stat3, and EGFR (**Supplementary Material**, mass spectrometry analysis). Considering that GP130 is an important signaling molecule for Stat3 activation (18), we further investigated the interactions of CDCA with EGFR, GRP78, GP130, and Stat3 by magnetic bead-immunoprecipitation assays. High levels of EGFR and Stat3 were detected in the proteins isolated from the magnetic bead precipitation samples, while GRP78 was not detected. GP130 was detected in magnetic bead supernatant, and magnetic bead precipitation samples, but at low levels, possibly because it may not be the actual binding protein, or may be non-specifically binding (**Figure 3E**). These results suggested that CDCA can act on HepG2 cells through protein binding.

To validate the above results, we repeated the experiment in *in vitro* without binding the magnetic beads and assayed the level of protein grabbed by the beads. The results (**Figure 3F**) showed that EGFR and Stat3 protein levels did not change significantly in HepG2 cells treated with CDCA plus sorafenib when compared to cells treated with either drug alone, but their phosphorylation decreased significantly after treatment with CDCA or CDCA plus sorafenib. GP130 protein levels also decreased after CDCA or CDCA plus sorafenib treatment. These results suggested that CDCA plus sorafenib may inhibit the proliferation and promote apoptosis of HepG2 cells *via* inhibiting EGFR and Stat3 signaling.

### CDCA Does Not Target Stat3 in HepG2 Cells

**Figure 4:**
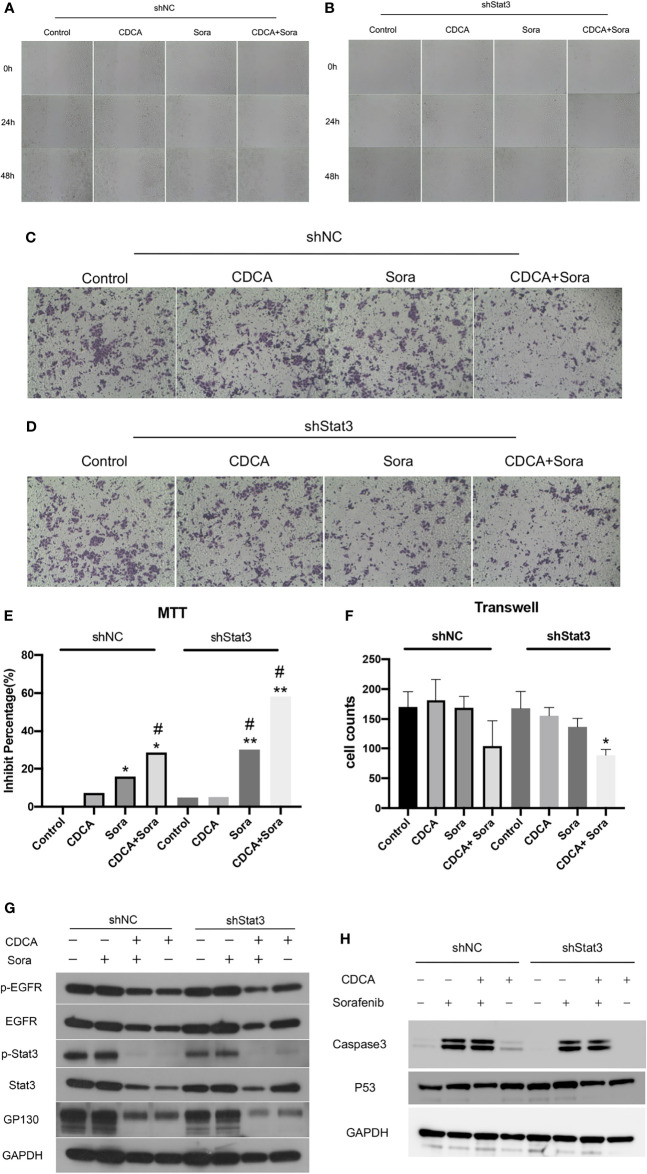
*In vitro* validation of the effect of CDCA on Stat3. After knockdown of cellular Stat3, intervention with the same dose of CDCA and sorafenib as before. **(A, B)** Results of cell scratch experiments before and after *Stat3* knockdown at 0, 24, and 48 h (10x). **(C, D)** Crystalline violet staining to observe cell proliferation before and after *Stat3* knockdown (20x). **(E)** MTT results. *P < 0.05 vs. shNC-Control, **P < 0.01 vs. shNC-Control, ^#^ *P* < 0.05 vs. shStat3-Control. **(F)** Transwell assay results. *P < 0.05 vs. shStat3-Control. **(G)** EGFR and STAT3 protein and phosphorylation levels before and after *Stat3* knockdown. **(H)** Effect of CDCA on apoptotic protein caspase 3 and p53 expression before and after *Stat3* knockdown.

**Figure 5:**
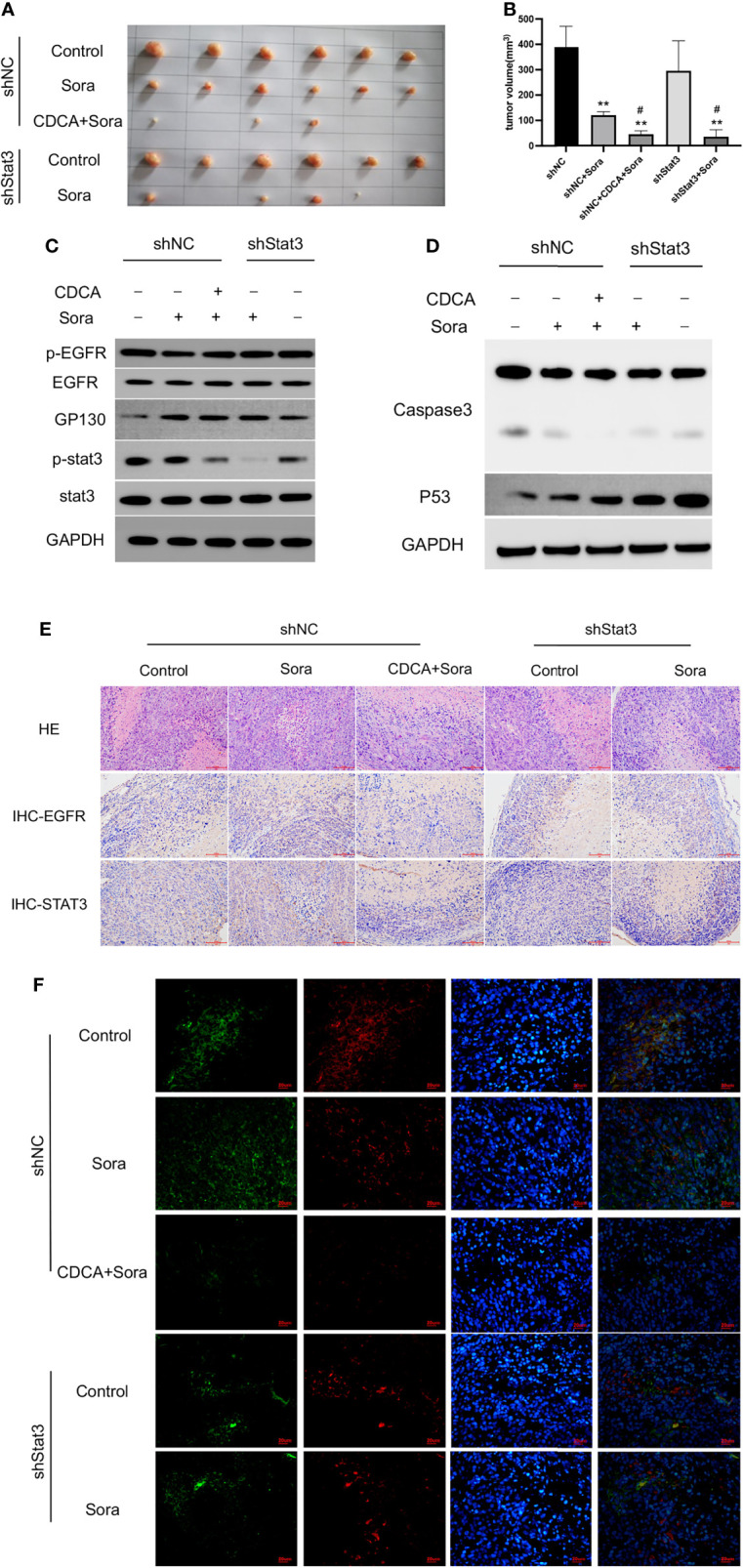
*In vivo* validation of the effect of CDCA on Stat3. The knockdown Stat3 cells were transplanted into nude mice, and the rest of the operation was performed as in previous *in vivo* experiments. **(A)** Transplanted tumors. **(B)** Transplanted tumor volumes. **P < 0.01 vs. shNC, ^#^ *P* < 0.01 vs. shStats. **(C)** EGFR and Stat3 protein and phosphorylation levels. **(D)** Caspase 3 and p53 protein expression. **(E)** HE and immunohistochemical staining of tumor tissues (20x). **(F)** Immunofluorescence staining of tumor tissues (40x).

Given that the EGFR/Stat3 signaling pathway is one of the classical pathways in tumor research and that EGFR is a key target of sorafenib, we were excited by the above findings. Therefore, we next investigated how CDCA modulates the EGFR/Stat3 pathway. Stat3 is one of a family of cytoplasmic transcription factors that have important roles in tumor proliferation, metastasis and drug resistance (19). Meanwhile, Stat3 phosphorylation can be promoted by EGFR (20). To verify whether CDCA could inhibit Stat3, we knocked down *Stat3* and repeated the *in vitro* and *in vivo* experiments. *In vitro* study, The combination of CDCA and sorafenib had an inhibitory effect on HepG2 cells, but Stat3 knockdown did not produce significant differences in cell migration, proliferation and invasion in each intervention group compared to the shNC groups (**Figures 4A–F**). After Stat3 knockdown, no significant differences were seen in p-EGFR, EGFR, p-Stat3, Stat3, gp130, caspase-3, and p53 protein expression in each experimental group compared to the shNC groups (**Figures 4G, H**). *In vivo* study, there’s no significantly change in tumor volume after Stat3 knockdown compared with shNC-Control group, but the tumor size decreased in the group of sorafenib plus shStat3 group compared with shStat3 group. The efficacy of CDCA combined with sorafenib was close to that of the sorafenib plus shStat3 group (**Figures 5A, B**). However, Stat3 knockdown did not show significant differences in the levels of EGFR, p-EGFR, Stat3, p-Stat3 and apoptosis proteins Caspase3 and p53 (**Figures 5C, D** and **S5**) Histology, immunohistochemistry, and immunofluorescence also showed no significant differences (**Figures 5E, F**).

### CDCA Enhances the Efficacy of Sorafenib by Inhibiting EGFR

**Figure 6:**
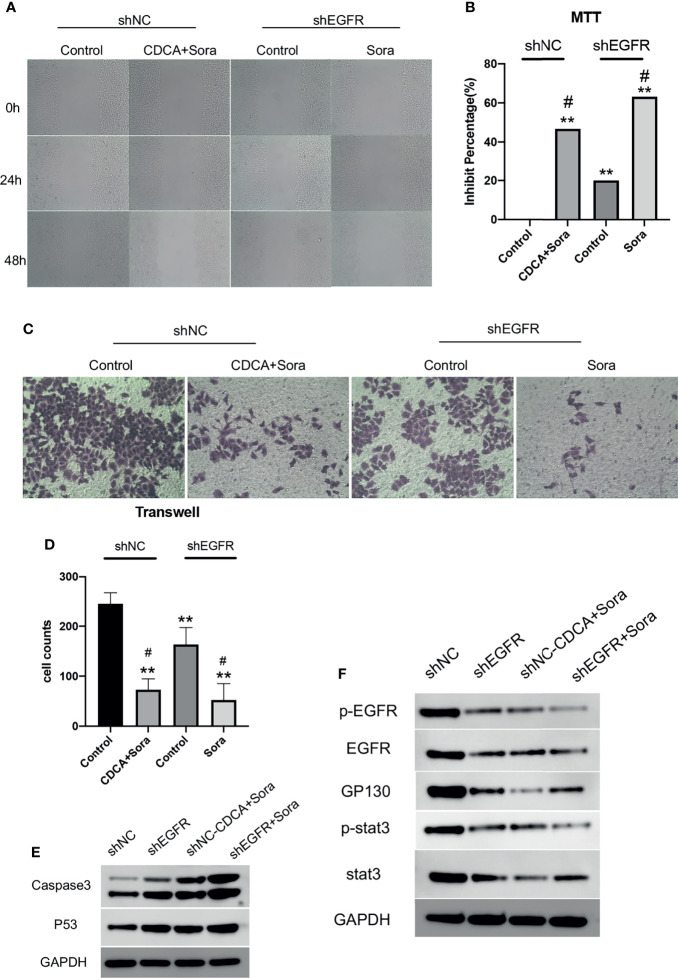
CDCA inhibits EGFR *in vitro*. After knockdown of cellular EGFR, intervention with the same dose of CDCA and sorafenib as before. **(A)** Cell scratch assays at 0, 24, and 48 h after intervention (10x). **(B)** MTT results. **P < 0.01 vs. shNC-Control, ^#^ *P* < 0.05 vs. shEGFR-Control. **(C)** Crystalline violet staining to observe cell proliferation before and after *EGFR* knockdown (20x). **(D)** Transwell assay results. **(E)** Effect of CDCA on apoptotic protein caspase 3 and p53 expression before and after *EGFR* knockdown. **(F)** EGFR and STAT3 protein and phosphorylation levels before and after *EGFR* knockdown.

Then we knocked down EGFR and repeated the *in vitro* and *in vivo* experiments. The results of scratch and MTT assays (**Figures 6A, B**) showed that *EGFR* knockdown enhanced the effect of sorafenib in inhibiting tumor cell migration and proliferation; migration was slower in the CDCA plus sorafenib group than in the control group and was comparable to that of cells treated with shEGFR followed by sorafenib. Invasion assays showed that the inhibitory effect of CDCA plus sorafenib was comparable to that of sorafenib plus shEGFR (**Figures 6C, D**). Western blot analyses (**Figures 6E, F** and **S6**) showed that the levels of EGFR, p-EGFR, Stat3, and p-Stat3 were reduced to different degrees in all treatment groups, whereas the expression of caspase 3 and p53 was increased. The above results indicated that CDCA plus sorafenib and *EGFR* knockdown followed by sorafenib exerted similar inhibitory effects on HCC cells. Taken together, the above findings suggested that CDCA can target and bind EGFR proteins on the cell surface, acting as an EGFR inhibitor.

**Figure 7:**
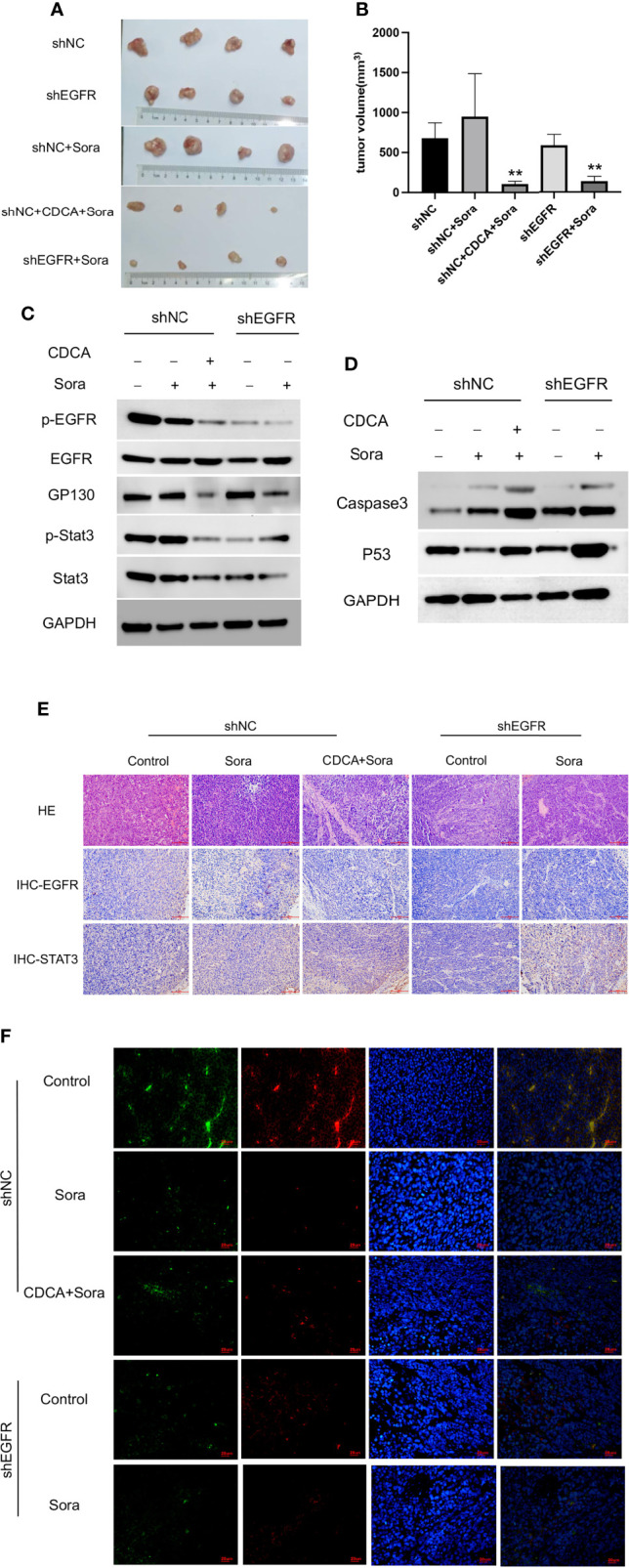
*In vivo* validation of CDCA-mediated inhibition of EGFR. The knockdown EGFR cells were transplanted into nude mice, and the rest of the operation was performed as in previous *in vivo* experiments. **(A)** Transplanted tumors. **(B)** Transplanted tumor volumes. **P < 0.01 vs. shNC-Sora. **(C)** EGFR and Stat3 protein and phosphorylation levels. **(D)** Caspase 3 and p53 protein expression. **(E)** HE and immunohistochemical staining of tumor tissues (20x). **(F)** Immunofluorescence staining of tumor tissues (40x).

*EGFR* knockdown HepG2 cells were inoculated into 4-week-old female Balb/c nude mice for validation of the above findings *in vivo*. The results showed significant tumor suppression in the CDCA plus sorafenib and shEGFR plus sorafenib groups (**Figures 7A, B**). Trends in the levels of EGFR, p-EGFR, Stat3, and p-Stat3 in transplanted tumors were consistent with the results obtained in the cell assays (**Figures 7C** and **S7**). Caspase 3 and p53 levels were increased in both the CDCA plus sorafenib and shEGFR plus sorafenib groups (**Figures 7D** and **S7**). Hematoxylin and eosin (HE) staining, immunohistochemical staining, and immunofluorescence staining of tumor tissues showed that CDCA plus sorafenib had a significant inhibitory effect on EGFR expression. These results suggested that CDCA inhibits EGFR protein expression *in vivo* to enhance the efficacy of sorafenib (**Figures 7E, F**).

### CDCA Binds to EGFR Extracellular Structural Domain to Target Hepatocellular Carcinoma Cells

**Figure 8:**
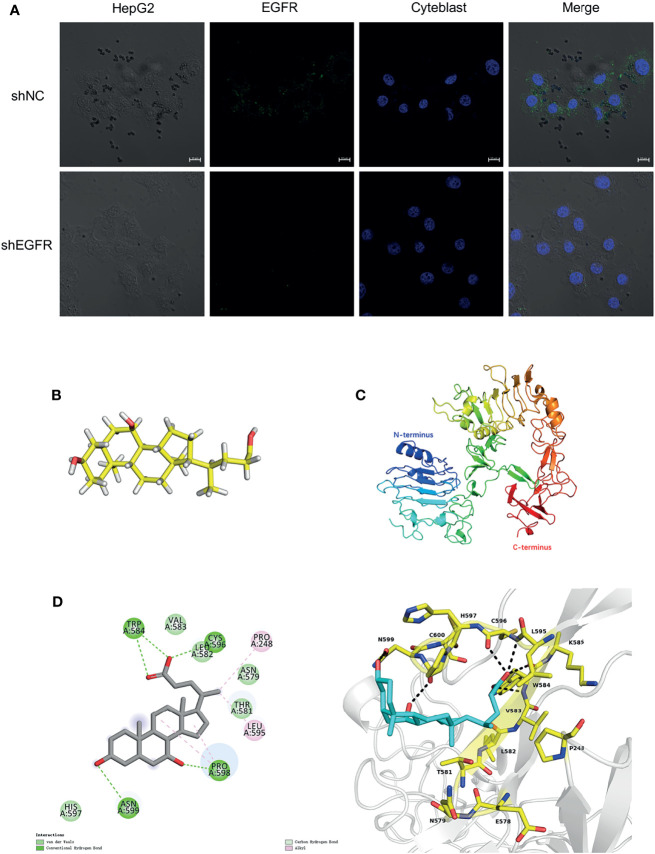
CDCA binds to EGFR extracellular structural domain. **(A)** Immunofluorescence confocal microscopy showing magnetic beads-CDCA aggregating around HepG2 cells (60x). **(B)** CDCA molecular structures. **(C)** EGFR protein 3D structures (ID:4UV7). **(D)** CDCA binds to the extracellular structural domain of EGFR.

To further verify the relationship between CDCA and EGFR, we performed fluorescence confocal experiments. When we knocked down *EGFR* in HepG2 cells, the aggregation of magnetic beads-CDCA around the cells decreased significantly (**Figure 8A**). In the control group, the beads did aggregate around HepG2 cells, corroborating that the aggregation depended on EGFR expression (**Figure 8A**). Simulated molecular docking techniques were used to investigate the sites where compounds bind to proteins (21). The structure of CDCA and EGFR is shown in the figure (**Figures 8B, C**). Predictive analysis using AMDOCK software revealed that CDCA binds to the extracellular structural domain of EGFR (**Figure 8D**). The above results provide ample evidence that CDCA enhances the effect of sorafenib by binding to EGFR and thus affecting the EGFR/Stat3 signaling pathway.

## Discussion

Liver cancer is one of the leading causes of death globally (1). How to effectively inhibit tumor cell proliferation currently is a hot research topic. Sorafenib, as a first-line drug for the treatment of liver cancer, has several side effects (22). It has been confirmed that sorafenib has good synergistic effects in combination with other drugs (23). However, it is necessary to explore more effective sorafenib combinations to improve patient outcomes.

In recent years, the potential anti-tumor effect of bile acids has been gradually noticed. For example, TUDCA, UDCA and LCA have been found to have anti-tumor activity (24–26). Although these results showed a potentiation effect in inhibiting tumor growth, we noted that the drug concentrations used in these studies exceeded the safe concentrations we explored (**Figure S1**). What’s more, we found there’s no significantly enhanced inhibition effect in HepG2 and SMMC 7721 cell lines by using 1ug/ml UDCA (within the safe concentration) combined with sorafenib (**Figure S8**), but it was found that CDCA could enhance the anti-tumor effect in the safe concentration range. Interestingly, CDCA is a major primary bile acid in humans. Phelan et al. (27) found that CDCA has a growth-inhibitory effect in various cancer cell models, whereas Liu et al. (28) found that CDCA is an oncogenic factor in non-small cell lung cancer. However, several studies have demonstrated that HS1200, a derivative of CDCA, has anti-tumor effects (29–33). The biological functions and specific mechanisms of CDCA remain to be investigated.

We investigated the effect of CDCA in combination with sorafenib on HepG2 cells and found that CDCA plus sorafenib showed significant inhibitory effects on HCC cells. Interestingly, we noted that in the formal experiment, the inhibition rate of HepG2 cells by 1ug/ml CDCA was close to 20%, and the inhibition rate of HepG2 cells by 1ug/ml CDCA plus sorafenib was close to 80% (**Figure 1A**), which were higher than that in the preliminary study (**Figure S2**). The reason we think is due to the difference between experimental batches, which is within the allowable range. *In vivo*, the combination treatment significantly inhibited the growth of transplanted tumors. We found that the potentiated effect of the drug combination may be related to the upregulation of pro-apoptotic proteins. Further, we found that CDCA could substantially accumulate around HepG2 cells using a magnetic beads-CDCA assay, suggesting that CDCA has HCC cell-targeting capacity. The isolation, identification, and validation of the binding proteins revealed that the target proteins were likely to be EGFR and STAT3.

EGFR (also known as HER-1), the expression product of proto-oncogene c-erbB-1, is closely related with tumor genesis and tumor development (34). High EGFR expression plays an important role in the onset and development of chronic liver disease and liver cancer (35–37), and can aggravate the invasiveness of liver cancer, while targeted inhibition of EGFR can reduce the invasiveness of cancer cells and exerts a certain effect in the treatment of liver cancer (38). At present, targeted therapies for EGFR are mainly divided into two categories (39). One category comprises tyrosine kinase inhibitors that can enter cells and compete with ATP to bind tyrosine kinase, indirectly inhibiting its function. The other category comprises anti-EGFR monoclonal antibodies, which mainly act on the extracellular domain of EGFR, competitively inhibiting the binding of various ligands (such as EGF and TGF-α) to the receptor and blocking its phosphorylation, which finally leads to the loss of EGFR activity, inhibition of tumor growth, and induction of apoptosis. EGFR is one of the targets of sorafenib.

As a downstream signal transducer and transcriptional activator of EGFR, STAT3 also has attracted substantial attention in relation to tumorigenesis and development (20). STAT3, a member of the STAT family, exists in the cytoplasm. STAT3 can bind to activated EGFR *via* its SH2 domain and phosphorylates its tyrosine at position 705 to form a dimer, which can be transferred into the nucleus to bind to certain DNA elements to regulate transcription (40). The excessive activation of STAT3 is closely related to the angiogenesis, invasion, and metastasis of various types of malignant tumors (41). Under normal physiological conditions, STAT3 activation can only be maintained for a short time, whereas STAT3 is continuously activated in various tumor cells (19).

Our findings suggest that CDCA may enhance the anti-tumor effects of sorafenib *via* inhibition of the EGFR/Stat3 signaling pathway. In *in vitro* and *in vivo* validation assays, the effect of CDCA plus sorafenib was comparable to that of sorafenib following *EGFR* knockdown, suggesting that CDCA can enhance the effect of sorafenib by binding to EGFR and inhibiting its activity. Although Stat3 protein was captured in the magnetic bead binding assay, no significant inhibition of Stat3 by CDCA was found in subsequent experiments. In fluorescence confocal experiments, we found that the aggregation of magnetic beads-CDCA around HepG2 cells was significantly reduced after *EGFR* knockdown, suggesting that CDCA exerts synergistic effects with sorafenib in HepG2 cells growth depending on EGFR protein. Molecular docking simulations validated that CDCA binds to the extracellular structural domain of EGFR. Therefore, we propose that CDCA enhances the efficacy of sorafenib by targeting binding to EGFR and thereby affecting the EGFR/Stat3 signaling pathway. CDCA may play the same role as an EGFR inhibitor in other tumors, and in preliminary experiments using other cell lines, CDCA was confirmed to similarly inhibit EGFR expression (**Supplementary Information**, **Figure S9**).

This study identified that CDCA enhances the anti-tumor effect of sorafenib and plays an inhibitory effect on EGFR. CDCA, as a bile acid, is an important endogenous metabolite in the human body and a major component of traditional Chinese medicines, such as bear bile powder, taurine, and pig bile powder. Our findings are expected to inspire future research on cancer treatment strategies.

## Conclusions

In conclusion, we confirmed that CDCA enhances the inhibitory effect of sorafenib on hepatocellular carcinoma cells by *in vivo* and *in vitro* experiments, which relies on the binding of CDCA to EGFR. This indicates that CDCA can act as an EGFR inhibitor and potentiate anti-tumor drugs effect. Since CDCA is a major bile acid secreted by the body, we are currently investigating more researches.

## Data Availability Statement

The original contributions presented in the study are included in the article/**Supplementary Material**. Further inquiries can be directed to the corresponding author.

## Ethics Statement

The animal study was reviewed and approved by The Experimental Animal Ethics Committee of CAIQ Health (TianJin) Inspection and Testing Co., LTD. (Approval No. 2020041501).

## Author Contributions

Y-LW perceived the idea, initiated the project, designed the experiments and was responsible for article revision. YangZ drafted the manuscript. YangZ performed all the cellular experiments including magnetic beads complex tests, molecular biology detection and some pathological detection. YanZ and X-JS were responsible for *in vivo* experiments and some pathological detection. J-XL, L-HW, and C-EX provided suggestions for the completion of the experiments and helped to draft the manuscript. All authors read and approved the final manuscript.

## Funding

This study was supported by the National Natural Science Foundation of China (grant No. 81503407) and the Fundamental Research Funds for the Central Universities (Scientific Research Innovation Team, grant No. 2019-JYB-TD004).

## Conflict of Interest

The authors declare that the research was conducted in the absence of any commercial or financial relationships that could be construed as a potential conflict of interest.

## Publisher’s Note

All claims expressed in this article are solely those of the authors and do not necessarily represent those of their affiliated organizations, or those of the publisher, the editors and the reviewers. Any product that may be evaluated in this article, or claim that may be made by its manufacturer, is not guaranteed or endorsed by the publisher.

## Abbreviations

CDCA, Chenodeoxycholic acid; HCC, Hepatocellular carcinoma; EGFR, Epidermal growth factor receptor.

## References

[1] Llovet JM Kelley RK Villanueva A Singal AG Pikarsky E Roayaie S. Hepatocellular Carcinoma. Nat Rev Dis Primers (2021) 7(1):6. doi: 10.1038/s41572-020-00240-3 33479224 PMC13537433

[2] Chidambaranathan-Reghupaty S Fisher PB Sarkar D. Hepatocellular Carcinoma (HCC): Epidemiology, Etiology and Molecular Classification. Adv Cancer Res (2021) 149:1–61. doi: 10.1016/bs.acr.2020.10.001 33579421 PMC8796122

[3] Cheng A-L Kang Y-K Chen Z Tsao C-J Qin S Kim JS. Efficacy and Safety of Sorafenib in Patients in the Asia-Pacific Region With Advanced Hepatocellular Carcinoma: A Phase III Randomised, Double-Blind, Placebo-Controlled Trial. Lancet Oncol (2009) 10(1):25–34. doi: 10.1016/S1470-2045(08)70285-7 19095497

[4] Llovet JM Montal R Sia D Finn RS. Molecular Therapies and Precision Medicine for Hepatocellular Carcinoma. Nat Rev Clin Oncol (2018) 15(10):599–616. doi: 10.1038/s41571-018-0073-4 30061739 PMC12452113

[5] Ardelt MA Fröhlich T Martini E Müller M Kanitz V Atzberger C. Inhibition of Cyclin-Dependent Kinase 5: A Strategy to Improve Sorafenib Response in Hepatocellular Carcinoma Therapy. Hepatol (Baltimore Md) (2019) 69(1):376–93. doi: 10.1002/hep.30190 PMC6590289 30033593

[6] Mao J Yang H Cui T Pan P Kabir N Chen D. Combined Treatment With Sorafenib and Silibinin Synergistically Targets Both HCC Cells and Cancer Stem Cells by Enhanced Inhibition of the Phosphorylation of STAT3/ERK/AKT. Eur J Pharmacol (2018) 832:39–49. doi: 10.1016/j.ejphar.2018.05.027 29782854

[7] Lachenmayer A Toffanin S Cabellos L Alsinet C Hoshida Y Villanueva A. Combination Therapy for Hepatocellular Carcinoma: Additive Preclinical Efficacy of the HDAC Inhibitor Panobinostat With Sorafenib. J Hepatol (2012) 56(6):1343–50. doi: 10.1016/j.jhep.2012.01.009 PMC3355195 22322234

[8] Dai N Ye R He Q Guo P Chen H Zhang Q. Capsaicin and Sorafenib Combination Treatment Exerts Synergistic Anti−Hepatocellular Carcinoma Activity by Suppressing EGFR and PI3K/Akt/mTOR Signaling. Oncol Rep (2018) 40(6):3235–48. doi: 10.3892/or.2018.6754 PMC6196646 30272354

[9] Wu L Feng J Li J Yu Q Ji J Wu J. The Gut Microbiome-Bile Acid Axis in Hepatocarcinogenesis. Biomed Pharmacother Biomed Pharmacother (2021) 133:111036. doi: 10.1016/j.biopha.2020.111036 33378947

[10] Joyce SA Gahan CGM. Bile Acid Modifications at the Microbe-Host Interface: Potential for Nutraceutical and Pharmaceutical Interventions in Host Health. Annu Rev Food Sci Technol (2016) 7:313–33. doi: 10.1146/annurev-food-041715-033159 26772409

[11] Fiorucci S Distrutti E. Chenodeoxycholic Acid: An Update on Its Therapeutic Applications. Handb Exp Pharmacol (2019) 256:265–82. doi: 10.1007/164_2019_226 31267167

[12] Han J Qin WX Li ZL Xu AJ Xing H Wu H. Tissue and Serum Metabolite Profiling Reveals Potential Biomarkers of Human Hepatocellular Carcinoma. Clin Chim Acta (2019) 488:68–75. doi: 10.1016/j.cca.2018.10.039 30389456

[13] Gong Y Zhang X Zhang Y Chu F Li G Zhang H Xu B. Bile Acids, Carriers of Hepatoma-Targeted Drugs? Pharmazie (2016) 71(3):139–45. doi: 10.1691/ph.2016.5120 27183708

[14] Liu G Kuang S Cao R Wang J Peng Q Sun C. Sorafenib Kills Liver Cancer Cells by Disrupting SCD1-Mediated Synthesis of Monounsaturated Fatty Acids the ATP-AMPK-mTOR-SREBP1 Signaling Pathway. FASEB J (2019) 33(9):10089–103. doi: 10.1096/fj.201802619RR 31199678

[15] Garten A Grohmann T Kluckova K Lavery GG Kiess W Penke M. Sorafenib-Induced Apoptosis in Hepatocellular Carcinoma Is Reversed by SIRT1. Int J Mol Sci (2019) 20(16):4048. doi: 10.3390/ijms20164048 PMC6719220 31430957

[16] Liu L Cao Y Chen C Zhang X McNabola A Wilkie D. Sorafenib Blocks the RAF/MEK/ERK Pathway, Inhibits Tumor Angiogenesis, and Induces Tumor Cell Apoptosis in Hepatocellular Carcinoma Model PLC/PRF/5. Cancer Res (2006) 66(24):11851–8. doi: 10.1158/0008-5472.CAN-06-1377 17178882

[17] Watanabe M Iizumi Y Sukeno M Iizuka-Ohashi M Sowa Y Sakai T. The Pleiotropic Regulation of Cyclin D1 by Newly Identified Sesaminol-Binding Protein ANT2. Oncogenesis (2017) 6(4):e311. doi: 10.1038/oncsis.2017.10 28368390 PMC5520487

[18] Selander KS Li L Watson L Merrell M Dahmen H Heinrich PC. Inhibition of Gp130 Signaling in Breast Cancer Blocks Constitutive Activation of Stat3 and Inhibits *In Vivo* Malignancy. Cancer Res (2004) 64(19):6924–33. doi: 10.1158/0008-5472.CAN-03-2516 15466183

[19] Zou S Tong Q Liu B Huang W Tian Y Fu X. Targeting STAT3 in Cancer Immunotherapy. Mol Cancer (2020) 19(1):145. doi: 10.1186/s12943-020-01258-7 32972405 PMC7513516

[20] Jackson NM Ceresa BP. EGFR-Mediated Apoptosis *via* STAT3. Exp Cell Res (2017) 356(1):93–103. doi: 10.1016/j.yexcr.2017.04.016 28433699 PMC5514375

[21] Pinzi L Rastelli G. Molecular Docking: Shifting Paradigms in Drug Discovery. Int J Mol Sci (2019) 20(18):4331. doi: 10.3390/ijms20184331 PMC6769923 31487867

[22] Abdel-Rahman O Lamarca A. Development of Sorafenib-Related Side Effects in Patients Diagnosed With Advanced Hepatocellular Carcinoma Treated With Sorafenib: A Systematic-Review and Meta-Analysis of the Impact on Survival. Expert Rev Gastroenterol Hepatol (2017) 11(1):75–83. doi: 10.1080/17474124.2017.1264874 27882800

[23] Romero D. Combination Set to Transform HCC Therapy. Nat Rev Clin Oncol (2020) 17(7):389. doi: 10.1038/s41571-020-0396-9 32457541

[24] Kim YH Kim JH Kim BG Lee KL Kim JW Koh S-J. Tauroursodeoxycholic Acid Attenuates Colitis-Associated Colon Cancer by Inhibiting Nuclear Factor kappaB Signaling. J Gastroenterol Hepatol (2019) 34(3):544–51. doi: 10.1111/jgh.14526 30378164

[25] Lee S Cho YY Cho EJ Yu SJ Lee J-H Yoon J-H. Synergistic Effect of Ursodeoxycholic Acid on the Antitumor Activity of Sorafenib in Hepatocellular Carcinoma Cells *via* Modulation of STAT3 and ERK. Int J Mol Med (2018) 42(5):2551–9. doi: 10.3892/ijmm.2018.3807 PMC6192782 30106087

[26] Trah J Arand J Oh J Pagerols-Raluy L Trochimiuk M Appl B. Lithocholic Bile Acid Induces Apoptosis in Human Nephroblastoma Cells: A Non-Selective Treatment Option. Sci Rep (2020) 10(1):20349. doi: 10.1038/s41598-020-77436-w 33230229 PMC7683553

[27] Phelan JP Reen FJ Dunphy N O’Connor R O’Gara F. Bile Acids Destabilise HIF-1α and Promote Anti-Tumour Phenotypes in Cancer Cell Models. BMC Cancer (2016) 16:476. doi: 10.1186/s12885-016-2528-2 27416726 PMC4946243

[28] Liu X Xue S Jiang H. CDCA Promotes Non-Small-Cell Lung Cancer (NSCLC) Migration by Regulating Akt/Erk1/2 Signaling Pathways. ERS International Congress 2019 Abstracts. (2019). p. PA4686.

[29] Choi YH Im EO Suh H Jin Y Yoo YH Kim ND. Apoptosis and Modulation of Cell Cycle Control by Synthetic Derivatives of Ursodeoxycholic Acid and Chenodeoxycholic Acid in Human Prostate Cancer Cells. Cancer Lett (2003) 199(2):157–67. doi: 10.1016/S0304-3835(03)00351-3 12969788

[30] Park SE Choi HJ Yee SB Chung HY Suh H Choi YH. Synthetic Bile Acid Derivatives Inhibit Cell Proliferation and Induce Apoptosis in HT-29 Human Colon Cancer Cells. Int J Oncol (2004) 25(1):231–6. doi: 10.3892/ijo.25.1.231 15202011

[31] Šarenac T Mikov M. Cervical Cancer, Different Treatments and Importance of Bile Acids as Therapeutic Agents in This Disease. Front Pharmacol (2019) 10:484. doi: 10.3389/fphar.2019.00484 31214018 PMC6558109

[32] Liu H Qin C-K Han G-Q Xu H-W Ren W-H Qin C-Y. Synthetic Chenodeoxycholic Acid Derivative, HS-1200, Induces Apoptosis of Human Hepatoma Cells *via* a Mitochondrial Pathway. Cancer Lett (2008) 270(2):242–9. doi: 10.1016/j.canlet.2008.05.014 18565645

[33] Park SE Lee SW Hossain MA Kim MY Kim M-N Ahn EY. A Chenodeoxycholic Derivative, HS-1200, Induces Apoptosis and Cell Cycle Modulation *via* Egr-1 Gene Expression Control on Human Hepatoma Cells. Cancer Lett (2008) 270(1):77–86. doi: 10.1016/j.canlet.2008.04.038 18554781

[34] Sigismund S Avanzato D Lanzetti L. Emerging Functions of the EGFR in Cancer. Mol Oncol (2018) 12(1):3–20. doi: 10.1002/1878-0261.12155 29124875 PMC5748484

[35] Badawy AA-G El-Hindawi A Hammam O Moussa M Gabal S Said N. Impact of Epidermal Growth Factor Receptor and Transforming Growth Factor-α on Hepatitis C Virus-Induced Hepatocarcinogenesis. APMIS (2015) 123(10):823–31. doi: 10.1111/apm.12431 26279457

[36] Tsai W-C Tsai W-C Lee H-S Jin J-S Gao H-W Chao T-K. Association Between Osteopontin and EGFR Expression With Clinicopathological Parameters in Hepatocellular Carcinoma. Chin J Physiol (2012) 55(6):412–20. doi: 10.4077/CJP.2012.BAA082 23286449

[37] Cuevas MJ Tieppo J Marroni NP Tuñón MJ González-Gallego J. Suppression of Amphiregulin/Epidermal Growth Factor Receptor Signals Contributes to the Protective Effects of Quercetin in Cirrhotic Rats. J Nutr (2011) 141(7):1299–305. doi: 10.3945/jn.111.140954 21562239

[38] Komposch K Sibilia M. EGFR Signaling in Liver Diseases. Int J Mol Sci (2015) 17(1):30. doi: 10.3390/ijms17010030 PMC4730276 26729094

[39] Singh D Attri BK Gill RK Bariwal J. Review on EGFR Inhibitors: Critical Updates. Mini Rev Med Chem (2016) 16(14):1134–66. doi: 10.2174/1389557516666160321114917 26996617

[40] Klampfer L. Signal Transducers and Activators of Transcription (STATs): Novel Targets of Chemopreventive and Chemotherapeutic Drugs. Curr Cancer Drug Targets (2006) 6(2):107–21. doi: 10.2174/156800906776056491 16529541

[41] Timme S Ihde S Fichter CD Waehle V Bogatyreva L Atanasov K. STAT3 Expression, Activity and Functional Consequences of STAT3 Inhibition in Esophageal Squamous Cell Carcinomas and Barrett’s Adenocarcinomas. Oncogene (2014) 33(25):3256–66. doi: 10.1038/onc.2013.298 23912451

