# Supplementary material for: Chenodeoxycholic Acid Enhances the Effect of Sorafenib in Inhibiting HepG2 Cell Growth Through EGFR/Stat3 Pathway
Source: Front Oncol. 2022 Feb 17;12:836333. doi: 10.3389/fonc.2022.836333 (PMC8891169; doi:10.3389/fonc.2022.836333)
Supplement: Supplementary file 1 [file DataSheet_1.docx]

**Supplementary Information**

Fig.S1. Inhibition effect by different drug concentrations of CDCA and UDCA using L02 cell line.

Fig.S2. Inhibition effect by CDCA plus sorafenib using HepG2 and SMMC 7721 cell lines.


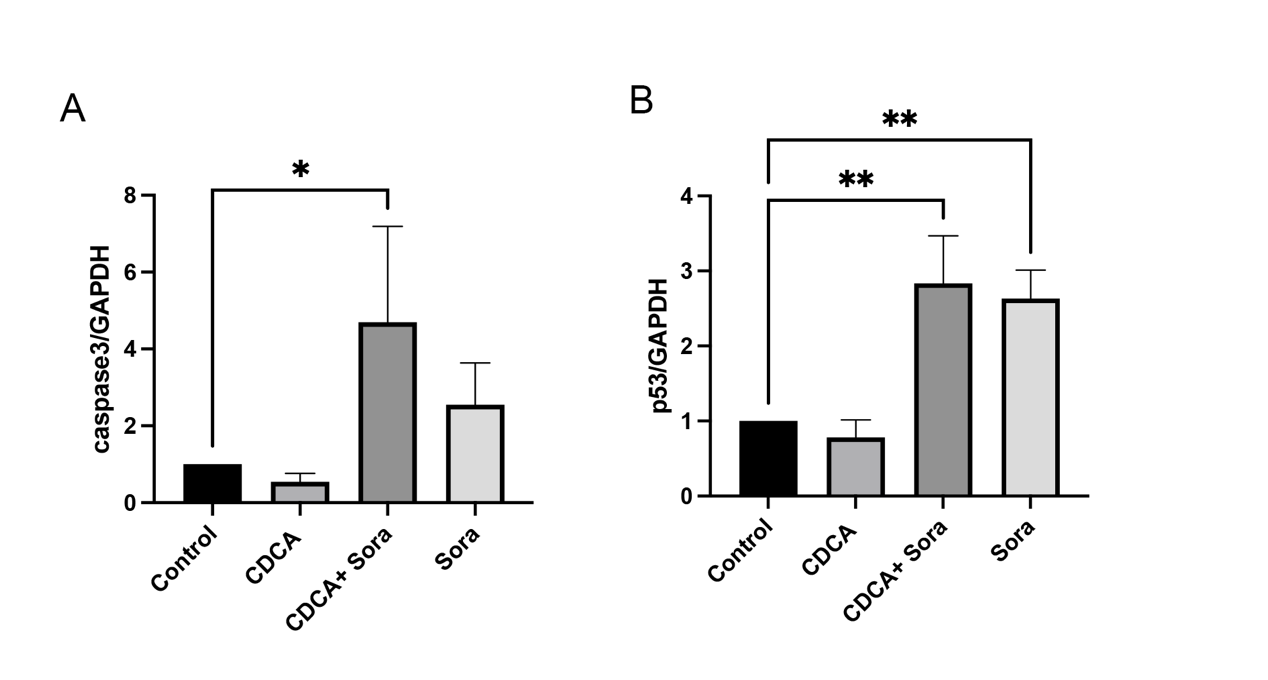


Fig.S3. WB quantitative analysis of Figure 1. **P* < 0.05, ***P* < 0.01.


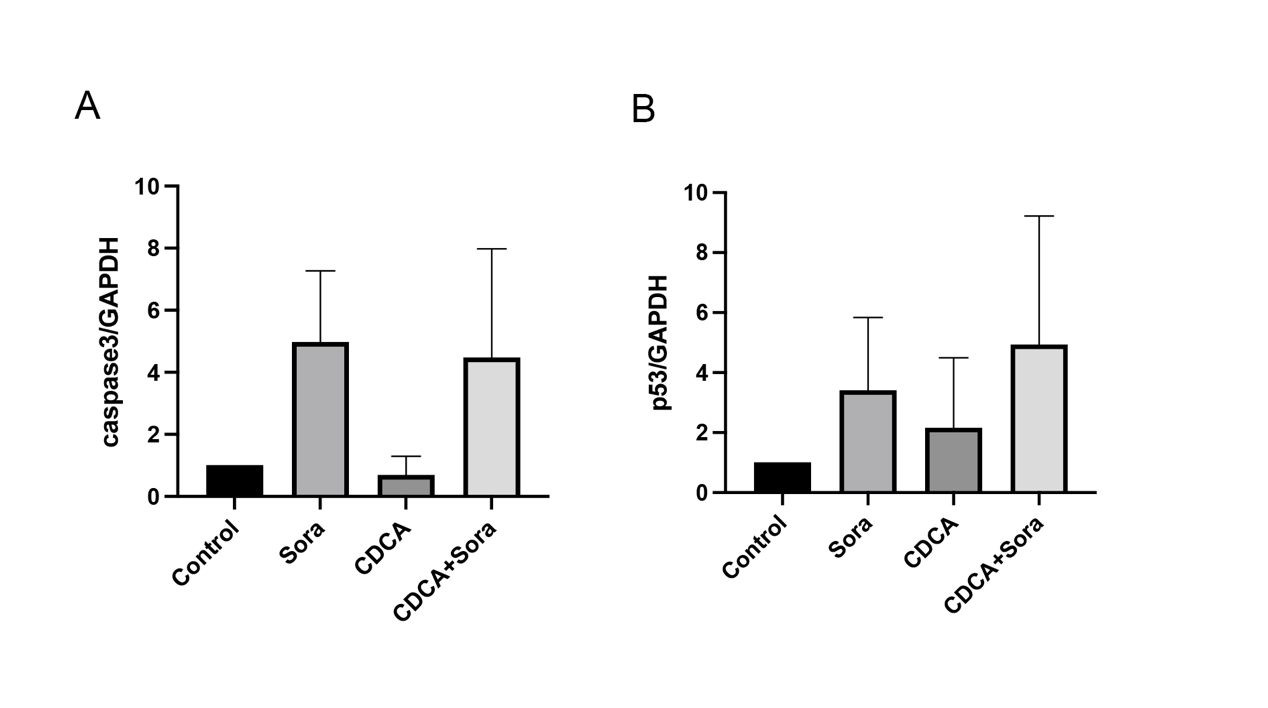


Fig.S4. WB quantitative analysis of Figure 2.


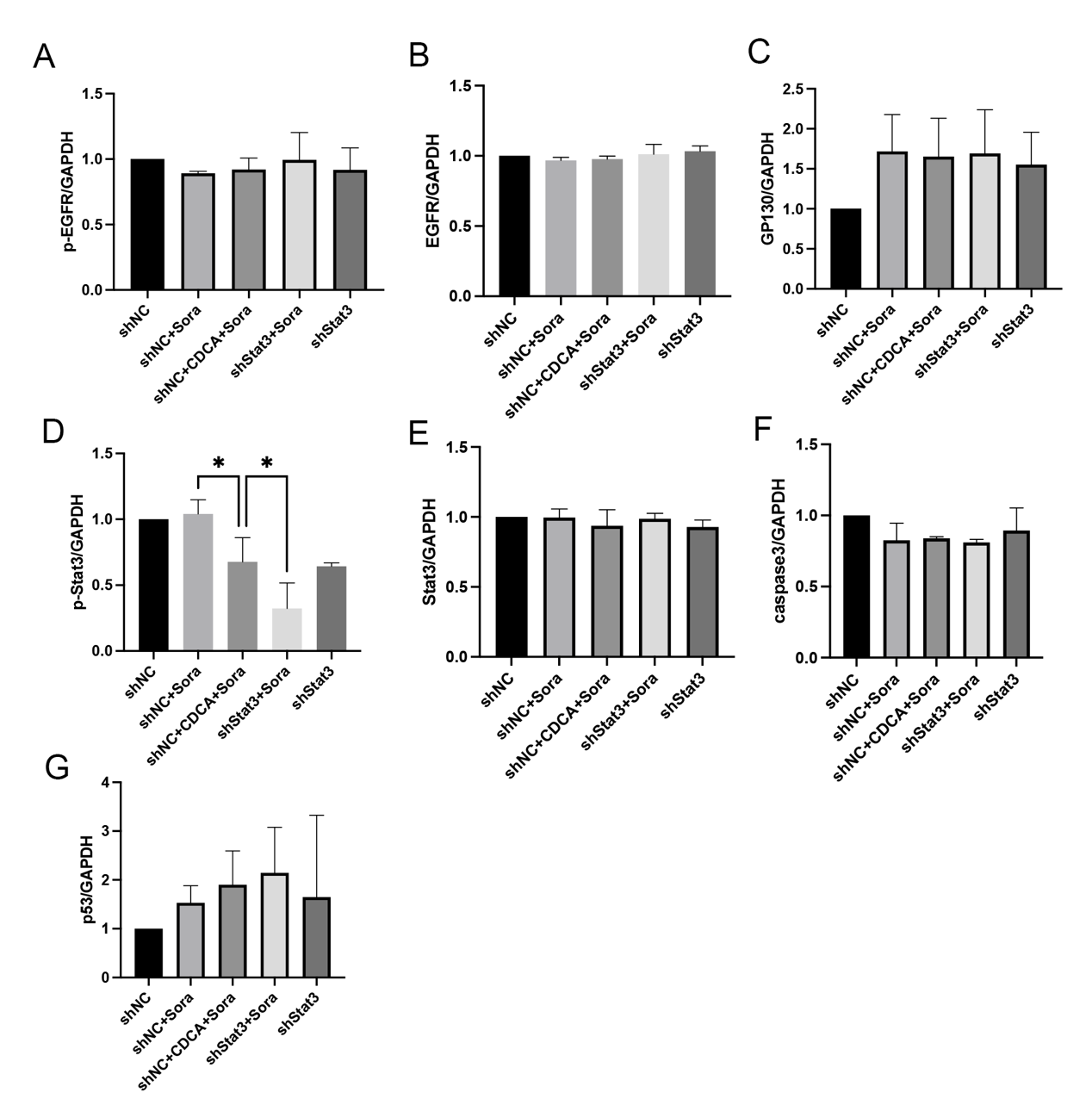


Fig.S5. WB quantitative analysis of Figure 5. **P* < 0.05.


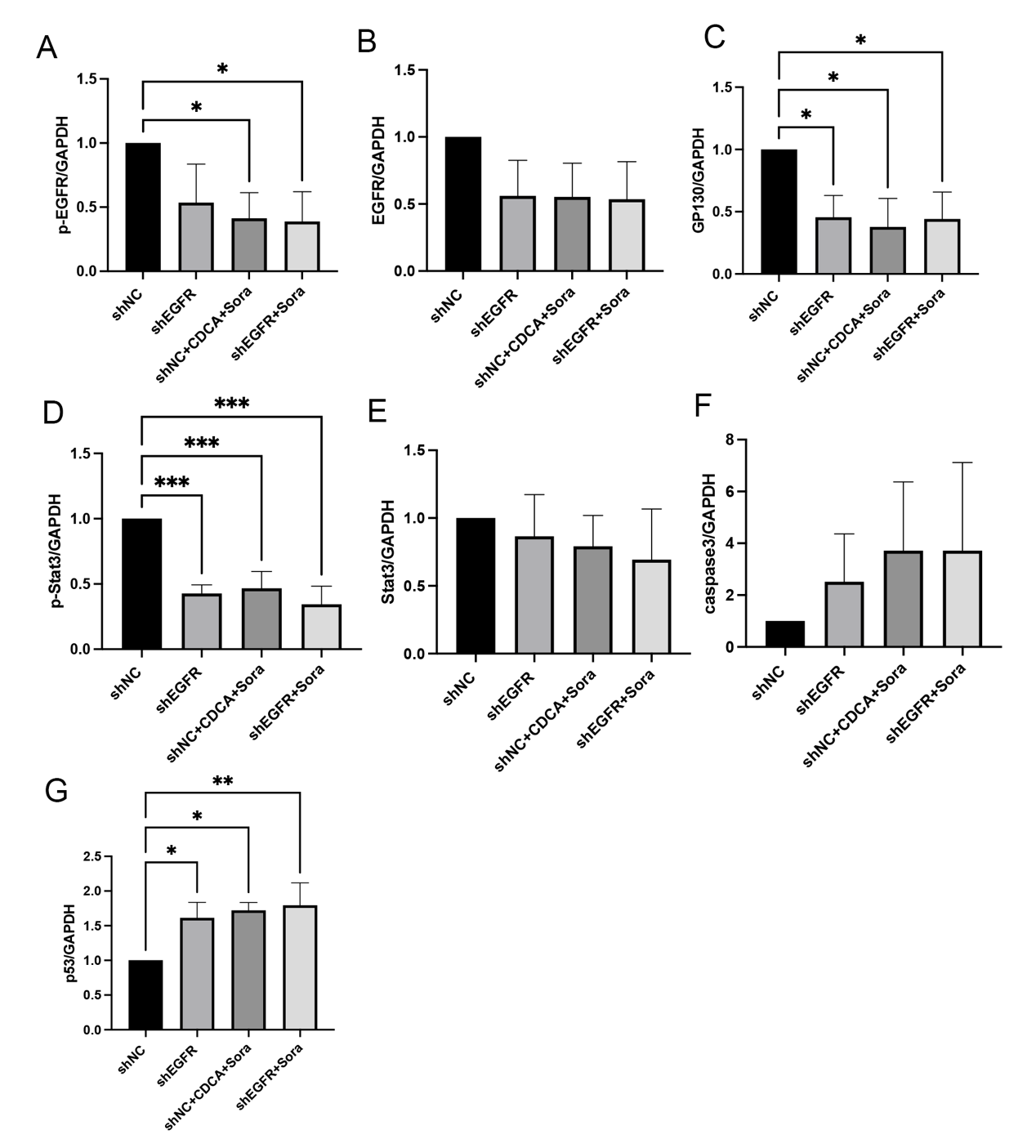


Fig.S6. WB quantitative analysis of Figure 6.**P* < 0.05 , ***P* < 0.01，****P* < 0.001.


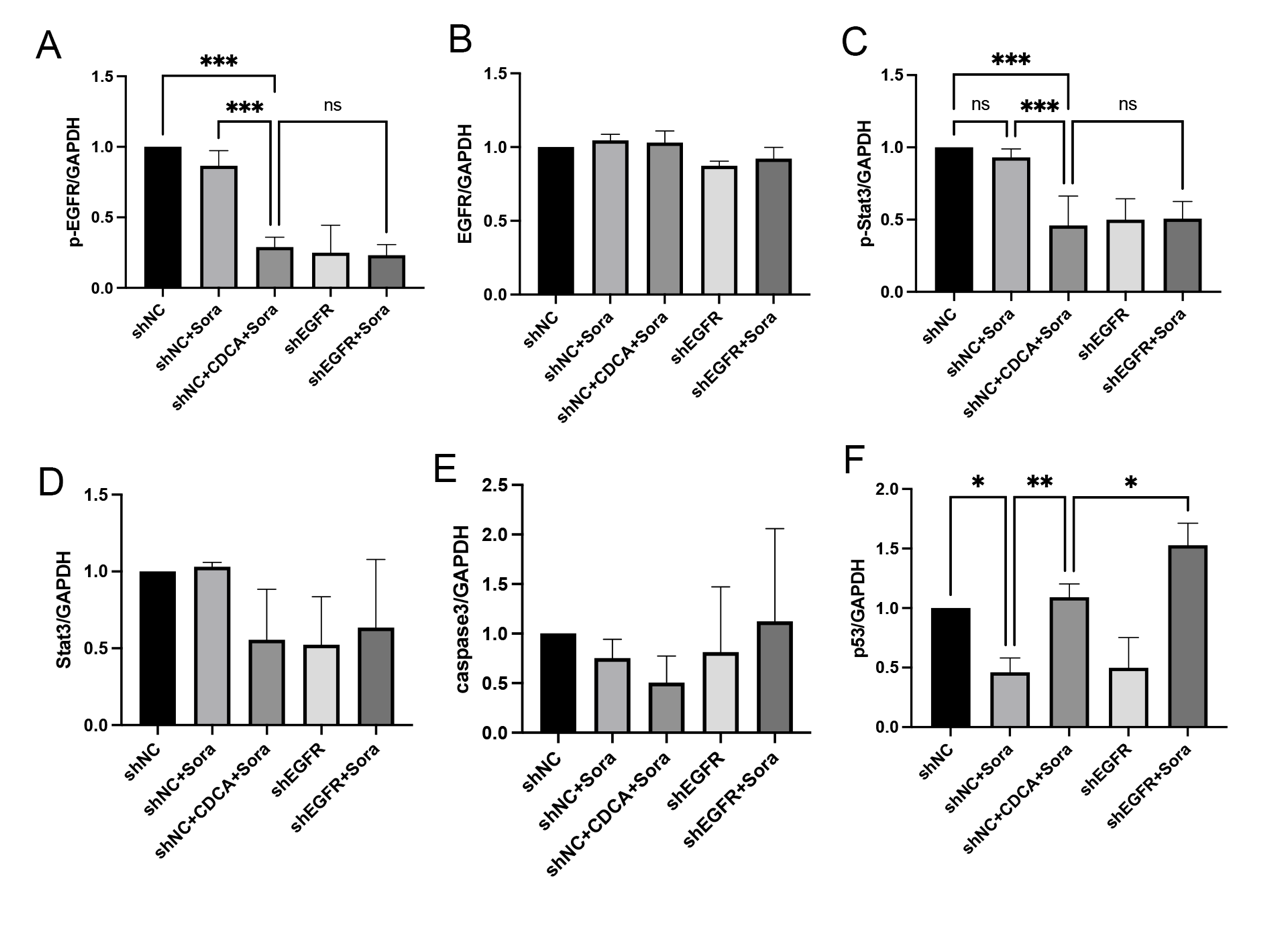


Fig.S7. WB quantitative analysis of Figure 7. ns: no significance,**P* < 0.05 , ***P* < 0.01，****P* < 0.001.

Fig.S8. Inhibition effect by UDCA plus sorafenib using HepG2 and SMMC 7721 cell lines.


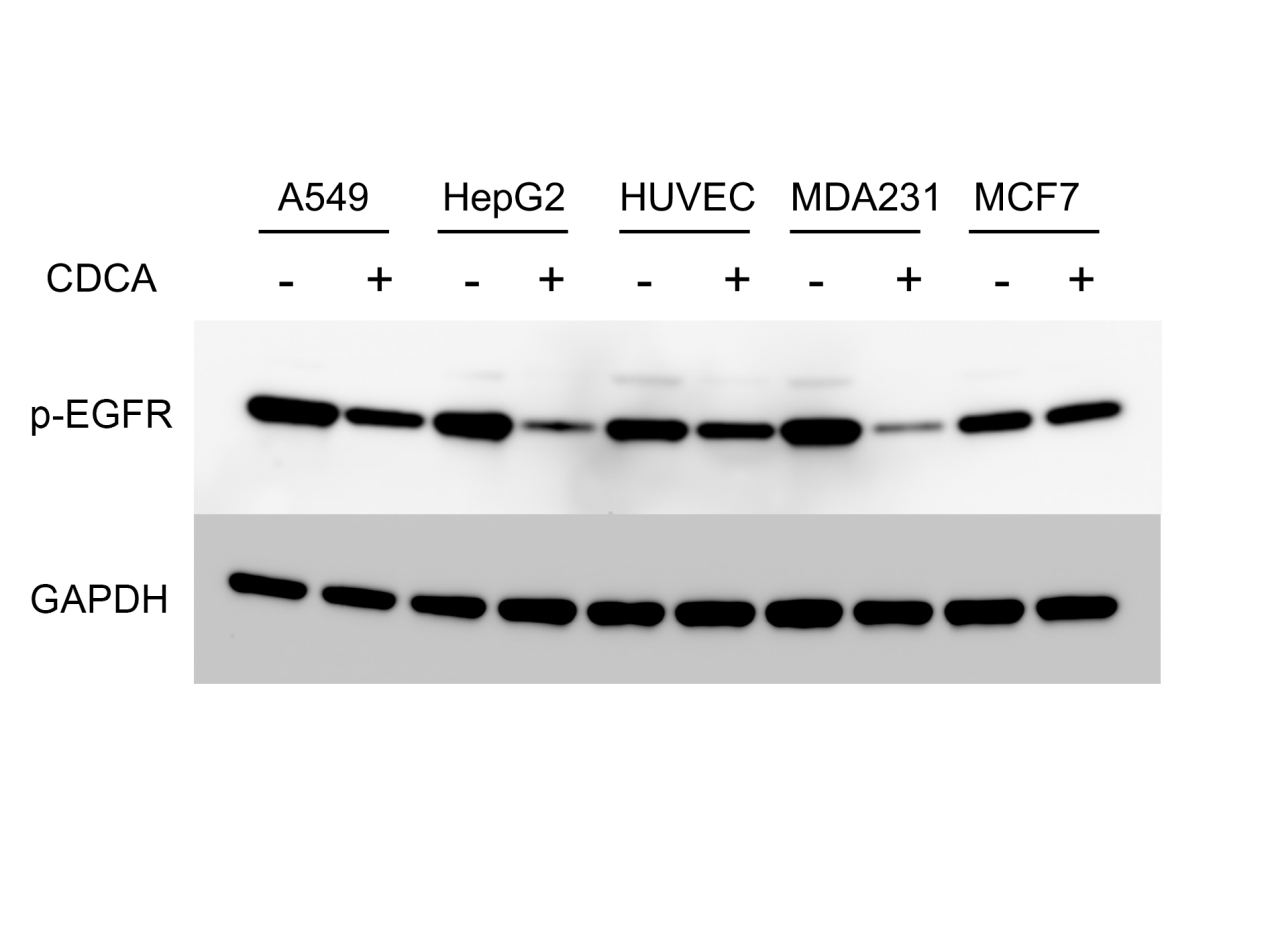


Fig. S9. CDCA inhibits EGFR expression in the indicated other tumor cell lines.
